# Suicidal behavior in patients with severe mental disorders prior to and during the COVID-19 pandemic

**DOI:** 10.1017/S003329172400299X

**Published:** 2024-12

**Authors:** Ellenor Mittendorfer-Rutz, Jakob Bergström, Pontus Josefsson, Heidi Taipale, Marit Sijbrandij, Anke Witteveen, Matteo Monzio Compagnoni, Antonio Lora, Mireia Felez-Nobrega, Josep Maria Haro, Maria Melchior, Judith van der Waerden, Katalin Gemes, Ridwanul Amin

**Affiliations:** 1 Department of Clinical Neuroscience, Division of Insurance Medicine, Karolinska Institutet, Stockholm, Sweden; 2 Department of Clinical, Neuro- and Developmental Psychology, VU University Amsterdam, WHO Collaborating Center for Research and Dissemination of Psychological Interventions, Amsterdam Public Health Research Institute, Amsterdam, the Netherlands; 3 Unit of Biostatistics, Epidemiology and Public Health, Department of Statistics and Quantitative Methods, University of Milano-Bicocca, Milan, Italy; 4 National Centre for Healthcare Research and Pharmacoepidemiology, University of Milano-Bicocca, Milan, Italy; 5 Department of Mental Health and Addiction Services, ASST Lecco, Lecco, Italy; 6 Research and Development Unit, Parc Sanitari Sant Joan de Déu, Institut Sant Joan de Déu-CERCA, Sant Boi de Llobregat, Spain; 7 Centre for Biomedical Research on Mental Health (CIBERSAM), Barcelona, Spain; 8 Departament de Medicina, Universitat de Barcelona, Barcelona, Spain; 9 Equipe de Recherche en Epidémiologie Sociale, Sorbonne Université, INSERM, Institut Pierre Louis d'Epidémiologie et de Santé Publique, Paris, France; 10 Division of Infectious Diseases, Department of Medicine, Karolinska Institutet, Stockholm, Sweden

**Keywords:** Covid-19 pandemic, mental disorder, suicide

## Abstract

**Background:**

Determining whether the incidence of suicidal behavior during the COVID-19 pandemic changed for those with severe mental disorders is essential to ensure the provision of suicide preventive initiatives in the case of future health crises.

**Methods:**

Using population-based registers, quarterly cohorts from the first quarter of 2018 (2018Q1) to 2021Q4 were formed including all Swedish-residents >10 years old. Interrupted time series and generalized estimating equations analyses were used to evaluate changes in Incidence Rates (IR) of specialised healthcare use for suicide attempt and death by suicide per 10 000 person-years for individuals with or without specific severe mental disorders (SMDs) during, compared to before the pandemic.

**Results:**

The IR (95% Confidence interval, CI) of suicide in individuals with SMDs decreased from 16.0 (15.0–17.1) in 2018Q1 to 11.6 (10.8–12.5) in 2020Q1 (i.e. the quarter before the start of the pandemic), after which it dropped further to 6.7 (6.3–7.2) in 2021Q2. In contrast, IRs of suicide attempt in SMDs showed more stable trends, as did the trends regarding suicide and suicide attempt for individuals without SMD. These discrepancies were most evident for individuals with substance use disorder and ASD/ADHD. Changes in IRs of suicide *v.* suicide attempt for one quarter during the pandemic for substance misuse were 11.2% *v.* 3.6% respectively. These changes for ASD/ADHD were 10.7% *v.* 3.6%.

**Conclusions:**

The study shows pronounced decreases in suicide rates in individuals with SMDs during the pandemic. Further studies aiming to understand mechanisms behind these trends are warranted to consult future suicide prevention strategies.

## Introduction

The Covid-19 pandemic is one of the most significant public health challenges of our time, associated with strong increases in mortality rates (Sanmarchi et al., 2021). Potential changes in mental health have been vividly discussed and could be attributable to social isolation measures affecting healthcare services, increasing labor market marginalization and disrupting individual and community support networks (Holmes et al., 2020). In addition to these stressors, suffering from a COVID-19 infection, death of close relatives, financial worries and a higher risk of domestic violence are other potentially triggering factors for poor mental health (Piquero, Jennings, Jemison, Kaukinen, & Knaul, 2021). Still, a recent systematic review reports no or minimal changes in mental health symptoms during as compared to before the pandemic (Sun et al., 2023).

In addition to potentially rising rates in mental ill-health, experts have also warned about an increase in suicidal behavior during the pandemic (Gunnell et al., 2020). Still, findings in the general population in up to 33 countries showed suicide rates to be primarily stable or lower-than-expected (Pirkis et al., 2021) and increases in only a few age and sex population strata (Pirkis et al., 2022). When it comes to suicide attempts, increased rates during the first year of the pandemic have, however, been reported in a meta-analysis including 54 studies (Dube, Smith, Sherry, Hewitt, & Stewart, 2021). In the mentioned papers primarily including studies on the general population, the authors emphasized the need for long-term follow-up studies including even the second year of the pandemic. Still, to date, there is limited knowledge concerning trends in both suicide attempt and suicide at the later stages of the pandemic.

Although suicidal behavior is known to have a multifactorial etiology, mental disorders are associated with the strongest risk (Bachmann, 2018). Compared to the general population, the risk of death by suicide is considerably higher in individuals with depressive and bipolar disorders (20–30 times) (Chesney, Goodwin, & Fazel, 2014; Miller & Black, 2020; Plans et al., 2019), schizophrenia (9 times) (Correll et al., 2022), Attention Deficit Hyperactivity Disorder/ADHD (7 times) (Septier, Stordeur, Zhang, Delorme, & Cortese, 2019), alcohol use disorder (2–3 times) (Isaacs et al., 2022), and post-traumatic stress disorder (2 times) (Akbar, Arya, Conroy, Wilcox, & Page, 2023). Moreover, a significant association between personality disorder (McClelland, Cleare, & O'Connor, 2023) and Autism Spectrum Disorder (ASD) with subsequent suicide was reported (Cleary et al., 2023). Given the additional stressors associated with the Covid-19 pandemic, these vulnerable groups may have been disproportionately affected regarding their risk for suicidal behavior. Moreover, despite efforts in compensating pandemic-related disruptions in mental health services by providing telemedicine services (WHO, 2020), individuals with mental disorders are likely to have been negatively affected by the changes in service provision, potentially increasing their risk of suicidal behavior (WHO, 2022; Witteveen et al., 2022).

Still, the current literature does not support any evidence of deteriorating mental health in individuals with mental disorders during the pandemic (Gemes et al., 2022; Robinson, Sutin, Daly, & Jones, 2022; Sun et al., 2023) and provides sparse information on how the pandemic has affected suicidal behavior among individuals with pre-existing mental disorders. The findings of the few available studies include higher rates of suicide attempt in psychiatric patients in general (Berardelli et al., 2021) and patients with bipolar disorder or schizophrenia in specific (Li et al., 2022). These studies suffer from methodological shortcomings such as limited geographical area, few assessment points and lack of access to longitudinal data. To date there is no available study included data on suicide.

Among the individuals with severe mental disorders (SMDs) there might be differences with regard to the degree of vulnerability regarding exposure to the pandemic. It can be hypothesized that individuals with specific SMDs such as non-affective psychosis and bipolar disorder, were disproportionally negatively affected by restrictions of social contacts as well as social and healthcare services. This diagnosis-specific information is highly warranted as suicide preventive initiatives are tailored towards specific mental disorders. Besides diagnosis-specific information, studies on rates of suicidal behavior over time need to consider several factors in the analyses, such as seasonal variations (Woo, Okusaga, & Postolache, 2012), differences in socio-economic status, living area and age (Bachmann, 2018) as well as work-related factors such as work disability and unemployment (Marlow, Xie, Tanner, Jo, & Kirby, 2021; Rahman, Alexanderson, Jokinen, & Mittendorfer-Rutz, 2016). Due to the known sex differences regarding both prevalence of mental disorders and suicidal behavior, any disparities in trends of suicidal behavior is of additional interest (Hawton & van Heeringen, 2009).

In order to address the current considerable knowledge gaps in the literature, this study aimed to investigate changes in incidence rates (IRs) of suicide attempt and suicide during as compared to before the COVID-19 pandemic in women and men with specific pre-existing SMDs in Sweden.

## Methods

### Study populations and data sources

The study populations were retrieved from de-identified register data which are available for each individual in Sweden. Retrospective and prospective data were individually linked using the following nationwide registers: (1) Longitudinal integrated database for health insurance and labor market studies (LISA) held by Statistics Sweden, including information on age, sex, country of birth, educational level, family situation, living area, employment status (Ludvigsson, Svedberg, Olén, Bruze, & Neovius, 2019); (2) Micro Data for Analysis of the Social Insurance database (MIDAS) held by Social Insurance Agency, including information on sickness absence and disability pension (Social Insurance Agency (Försäkringskassan), 2011); (3) National Patient Register (NPR) held by the National Board of Health and Welfare, which includes date and diagnoses of in- and specialized outpatient care (Forsberg et al., 2009; Ludvigsson et al., 2011); and (4) Cause of Death register (CDR), providing information on date and cause of death held by the National Board of Health and Welfare (Brooke et al., 2017). The observation period for the study consisted of quarterly periods from the 1st quarter of 2018 (2018Q1) up to 2021Q2 for suicide and up to 2021Q4 for suicide attempt. All individuals, >10 years old, residing in Sweden at the start of a quarter and during the 365 days preceding the start of the quarter were included and formed the study population for that quarter.

### Outcome measures

Specialized healthcare use for a suicide attempt and death by suicide as two separate outcome measures were defined based on the codes of the International Classification of Diseases version 10 (ICD-10) X60–X84 and Y10–Y34 in the NPR and CDR, respectively. Events of undetermined intent (ICD-10: Y10–Y34) were also included to limit the potential effects of underreporting and regional and temporal variations in case ascertainment (Linsley, Schapira, & Kelly, 2001; Runeson, Haglund, Lichtenstein, & Tidemalm, 2016) In Sweden, a high validity of the cause-of-death assessment process is guaranteed through a mandatory reporting system and a high coverage of forensic autopsies (Brooke et al., 2017).

### Pre-existing severe mental disorders

Pre-existing SMDs were identified based on main and side diagnoses in inpatient and specialized outpatient healthcare during the 365 days before the start of each observed quarter. Once identified by that way, individuals remained in the exposed group (i.e. specific mental disorder) in the following quarters. Seven groups of pre-existing SMDs were identified using ICD-10 codes: Substance misuse (F10–F19); Non-affective psychosis (F20–F29); Depressive/bipolar disorder (F30–F34); Stress- & neurotic-related disorders (F40–F48); Personality disorder (F60–F69); ASD/ADHD (F84, F90); Other mental disorders (other F-codes than the ones mentioned for the specific diagnostic groups, including organic mental disorders, persistent mood disorders, behavioral syndromes, mental retardation and disorders of the psychological development). A reference cohort of individuals without pre-existing SMDs was also created. The reference group did not have any ICD-10 F-codes as main or side diagnosis in inpatient or specialized outpatient healthcare during the 365 days before the start of the first observed quarter in 2018 (and for subsequent quarters, not in any of the respective time periods preceding the start of the respective quarter).

### Covariates

The following socio-demographic and work-related variables were considered in the multivariable analyses: sex; age; educational level; family situation; living area; country of birth; days with unemployment during the calendar year preceding the year of observation; net days of sickness absence; disability pension. Sex and age were measured in the year of each observation quarter. Other socio-demographic variables were measured at the end of each year preceding the respective observation year. Unemployment was measured during the calendar year preceding the year of observation. Other work-related variables were measured during the 365 days before the start of each observed quarter. Table 1 shows the categories of all covariates.

**Table 1.** Descriptive statistics of the study population (*N* = 8 741 608) in the first quarter of observation (January to March 2018)

Characteristics^a^	No pre-existing severe mental disorder^b^ *n* (column %)	Pre-existing severe mental disorder^b^ *n* (column %)
All, *n* (row %)	8 294 643 (94.9)	446 965 (5.1)
Sex		
Women	4 135 723 (49.9)	236 220 (52.8)
Men	4 158 920 (50.1)	210 745 (47.2)
Age groups (years)		
11–19	917 852 (11.1)	71 979 (16.1)
20–25	655 044 (7.9)	48 729 (10.9)
26–35	1 266 142 (15.3)	77 577 (17.4)
36–45	1 179 115 (14.2)	60 534 (13.5)
46–55	1 264 977 (15.3)	59 339 (13.3)
56–65	1 094 302 (13.2)	47 892 (10.7)
66-	1 917 211 (23.1)	80 915 (18.1)
Educational level (years)		
Elementary (0–9)	2 144 825 (25.9)	163 983 (36.7)
High school (10–12)	3 293 207 (39.7)	172 094 (38.5)
University/college (>12)	2 723 873 (32.8)	99 516 (22.3)
Missing information	132 738 (1.6)	11 372 (2.5)
Family situation		
Married or cohabitant without children	2 036 377 (24.6)	56 835 (12.7)
Married or cohabitant with children	1 701 964 (20.5)	46 352 (10.4)
Single without children	4 300 811 (51.9)	323 222 (72.3)
Single with children	255 491 (3.1)	20 556 (4.6)
Living area		
Cities	3 214 061 (38.7)	185 944 (41.6)
Towns and suburbs	3 435 264 (41.4)	180 347 (40.3)
Rural areas	1 645 318 (19.8)	80 674 (18.0)
Country of birth		
Swedish-born	6 713 756 (80.9)	378 029 (84.6)
Foreign-born	1 580 887 (19.1)	68 936 (15.4)
Unemployment^b^ (days)		
1–180	382 123 (4.6)	40 266 (9.0)
181–365	125 125 (1.5)	10 158 (2.3)
Disability pension (yes)	252 869 (3.0)	79 783 (17.8)
Sickness absence^b^ (days)		
1–90	390 170 (4.7)	37 125 (8.3)
91–180	71 092 (0.9)	17 278 (3.9)
181–365	67 165 (0.8)	41 820 (9.4)
Pre-existing severe mental disorder^b^		
Substance misuse disorder	–	73 874 (16.5)
Non-affective psychosis	–	34 202 (7.7)
Depression/Bipolar disorder	–	132 756 (29.7)
Stress & neurotic related disorders	–	159 587 (35.7)
Personality disorder	–	25 333 (5.7)
ASD/ADHD^c^	–	121 770 (27.2)
Other mental disorders	–	95 007 (21.3)

a All characteristics were measured on 31 December 2017 except age which is based on the age in the year of the observed quarter, i.e. 2018.

b Measured during the entire year preceding year of observation, i.e. 2017.

c Autism spectrum disorders/Attention deficit hyperactivity disorders.

### Data analysis

Interrupted time series (ITS) was the chosen design to evaluate changes in Incidence Rates (IR) per 10 000 person-years and 95% Confidence intervals (CI) during pre- and pandemic periods regarding death by suicide or suicide attempts. We considered 2020Q2 (April–June) as the beginning of the Covid-19 pandemic period because the World Health Organization declared the start of the pandemic on 2020-03-11. Therefore, we used this quarter as our change point to define pre- and pandemic time periods yielding nine quarters with pre- and five quarters with Covid-19 pandemic. We used IRs defined as the number of cases experiencing the event (death by suicide or suicide attempt) per quarter in the nominator and follow-up time (days observed per quarter) in the denominator considering the incomplete follow-up times due to death, migration or individuals included in the population after baseline.

As the outcomes are count variables, a log-linear Poisson regression model with follow-up time as an offset variable was performed using Generalized Estimating Equations (GEE) with robust standard errors (Wagner, Soumerai, Zhang, & Ross-Degnan, 2002). Time was entered into the model as two continuous variables. The first ‘time’ variable had the values 1,2,3…,14 for the death by suicide outcome (16 quarters for the outcome suicide attempts) and was an estimate of the change in IR per quarter pre-pandemic. The second time variable had the value zero up to the change point (9th quarter) and thereafter a count with an increase of one. It estimated the difference in the changes in IRs between pre- and pandemic periods. To extract the changes in IRs during COVID-19 pandemic, we added the coefficients for the two ‘time’ variables. The mathematical basis for this procedure is provided in the online Supplementary material. For interpretation of the findings, if the change in IR during the pandemic was e.g. 0.89, there was an 11% change per quarter during this period.

As ITS is a within-individual analysis, our main analysis was to estimate the change in IR for the two time periods and then estimate the ratio of change in IRs using GEE. This was implemented for each mental disorder separately. We also performed an analysis including sex in the model as an effect modifier by adding interaction terms between sex and the two ‘time’ variables yielding different regression lines for the sexes. As the occurrence of death by suicide underlies seasonal variation, a ‘seasonal’ component was added to the crude and adjusted models (not for the suicide attempt analysis) as a Fourier transformation of time with a pair of cosine and sinus functions, cosine (2 × pi × time)/and sinus (pi × time)/4 (Wagner et al., 2002) (online Supplementary material).

For Poisson regression models it does not matter if data are structured on individual or aggregated level. Hence, we aggregated the data to get the number of deaths by suicide, number of any suicide attempt and number of days observed by quarter prior to the GEE analyses. In the analysis with sex as an effect modifier, we aggregated the data by sex as well. Sensitivity analyses are described in the online Supplementary material.

Data management was performed using Stata (version 17) and analyses using R (version 4.1.3, ‘*geepack*’ package).

### Sensitivity analyses

In our ITS analysis we assumed that the studied population did not exhibit significant demographical changes which can be related to our outcome measures. We evaluated this assumption by performing a sensitivity analysis adjusting for time-dependent variables in the GEE model. We also included fixed variables (sex, age) as it can be interesting to see the results for the mental disorder groups as if they had the same demographical distribution. Another sensitivity analysis was performed including only deaths by suicide (X60–84) as the outcome; to validate if the same patterns could be reproduced compared to the outcome including the ‘events of undetermined intent’ (Y10–34). Similar sensitivity analysis was performed for the outcome of suicide attempts with the exception that additional data was available for up to 2021Q4 and no seasonality was assumed. To address the potential competing risk of death due to Covid-19 among older individuals, another set of sensitivity analyses was carried out excluding those over 66 years.

## Results

Descriptive statistics of the study population in 2018Q1 comprising 8 741 608 individuals of which 446 965 (5%) individuals had a pre-existing SMD are reported in Table 1

### Suicide

**Figure 1.**
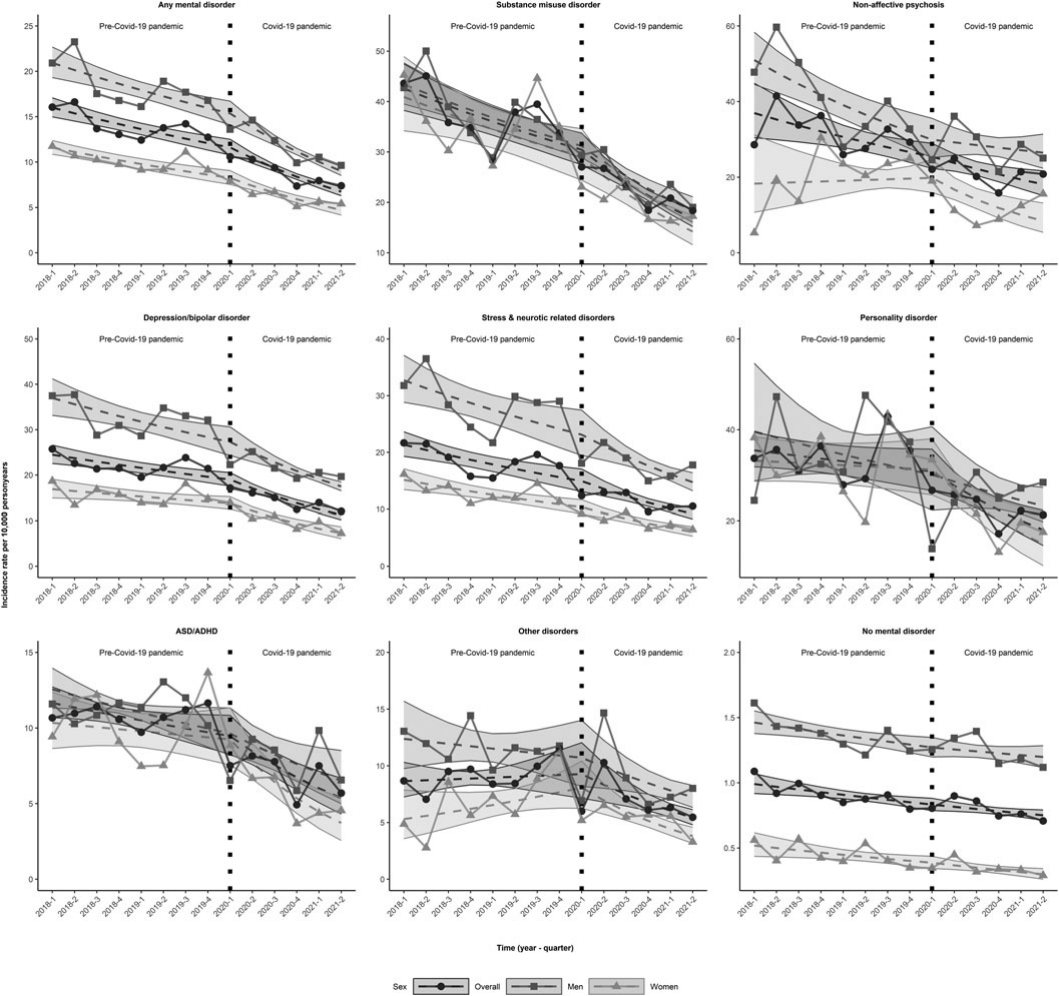
Crude observed and estimated incidence rates of suicide per 10 000 person-years with 95% confidence intervals during the years preceding the start of the Covid-19 pandemic (first quarter 2018 or 2018Q1–2020Q1) and the years after start of the pandemic (2020Q2–2021Q2) for individuals with specific severe mental disorders treated in specialized healthcare and individuals without such treated disorders the year preceding the start of the respective quarter, covering the entire population > 10 years of age in Sweden, stratified by sex; ASD/ADHD, Autism spectrum disorders/Attention deficit hyperactivity disorders.

The IR (95% CI) per 10 000 person-years of suicide in individuals with any pre-existing SMD decreased from 16.0 (15.0–17.1) in 2018Q1 to 11.6 (10.8–12.5) in 2020Q1 (i.e. the quarter before the start of the pandemic), after which it dropped further to 6.7 (6.3–7.2) in 2021Q2 (Fig. 1 and online Supplementary Table S1). These trends were similar across the sexes with men generally having twice the IR of women in any specific quarter. Considering specific SMDs, individuals with substance misuse (42.6; 38.2–47.5) and non-affective psychosis (36.9; 30.4–44.8) had the highest IRs; 95% CIs in 2018Q1.

Before the pandemic, IRs of suicide decreased slightly (range: 2%-5%) or kept stable for all pre-existing SMD diagnostic groups (Fig. 1, Table 2, online Supplementary Table S1). During the pandemic, however, suicide rates decreased considerably (range: 7–11%) for all SMD groups (Table 2). Strongest decreases in IRs of suicide during the pandemic were seen in individuals with substance misuse disorder (11.2% decrease for every quarter change), ASD/ADHD (10.7%) and personality disorder (10.2%) (Table 2). The degree of change in suicide rates during the pandemic compared to the pre-pandemic period was strongest among those with personality disorder and ASD/ADHD (Ratio of change in IR 0.914; 0.85–0.99 and 0.916; 0.85–0.98, respectively). IRs of suicide rates remained relatively stable for individuals without pre-existing SMDs both before and during the pandemic (Table 2).

**Table 2.** Change in crude Incidence Rates (IR)^a^ before (first quarter 2018 or 2018Q1–2020Q1) and during the Covid-19 pandemic (2020Q2–2021Q2) and the ratio of change in IRs during *v.* before the pandemic^b^ of suicide for groups of individuals with/without pre-existing severe mental disorders treated in specialised healthcare the year preceding the respective quarter

	Before Covid-19 pandemic		During Covid-19 pandemic		During v. before Covid-19 pandemic	
Pre-existing severe mental disorder	Change in IR (95% CI)	*p* value	Change in IR (95% CI)	*p* value	Ratio of change in IR (95% CI)	*p* value
Any mental disorders	0.96 (0.95–0.97)	<0.001	0.89 (0.87–0.92)	<0.001	0.93 (0.90–0.97)	<0.001
Substance misuse disorder	0.96 (0.94–0.98)	<0.001	0.89 (0.86–0.92)	<0.001	0.93 (0.88–0.97)	0.002
Non-affective psychosis	0.95 (0.92–0.99)	0.006	0.93 (0.89–0.98)	0.011	0.98 (0.91–1.06)	0.587
Depression/Bipolar disorder	0.97 (0.95–0.98)	<0.001	0.90 (0.87–0.93)	<0.001	0.93 (0.89–0.97)	<0.001
Stress & neurotic-related disorders	0.96 (0.93–0.98)	<0.001	0.91 (0.87–0.94)	<0.001	0.95 (0.89–1.01)	0.078
Personality disorder	0.98 (0.96–1.00)	0.101	0.89 (0.84–0.96)	0.001	0.91 (0.85–0.99)	0.023
ASD/ADHD^c^	0.97 (0.95–0.99)	0.037	0.89 (0.84–0.94)	<0.001	0.92 (0.85–0.98)	0.018
Other mental disorders	1.01 (0.96–1.06)	0.741	0.89 (0.84–0.96)	0.001	0.89 (0.79–0.99)	0.045
No mental disorders	0.98 (0.96–0.993)	0.003	0.98 (0.96–0.99)	0.024	1.00 (0.97–1.03)	0.947

a Slopes based on the GEE models with a linear fit showing the % change in IR for one unit (quarter) change during the pre- and pandemic periods.

b Estimates of slopes during the pandemic divided by the estimates of slopes before the pandemic.

c Autism spectrum disorders/Attention Deficit Hyperactivity Disorders.

### Suicide attempt

**Figure 2.**
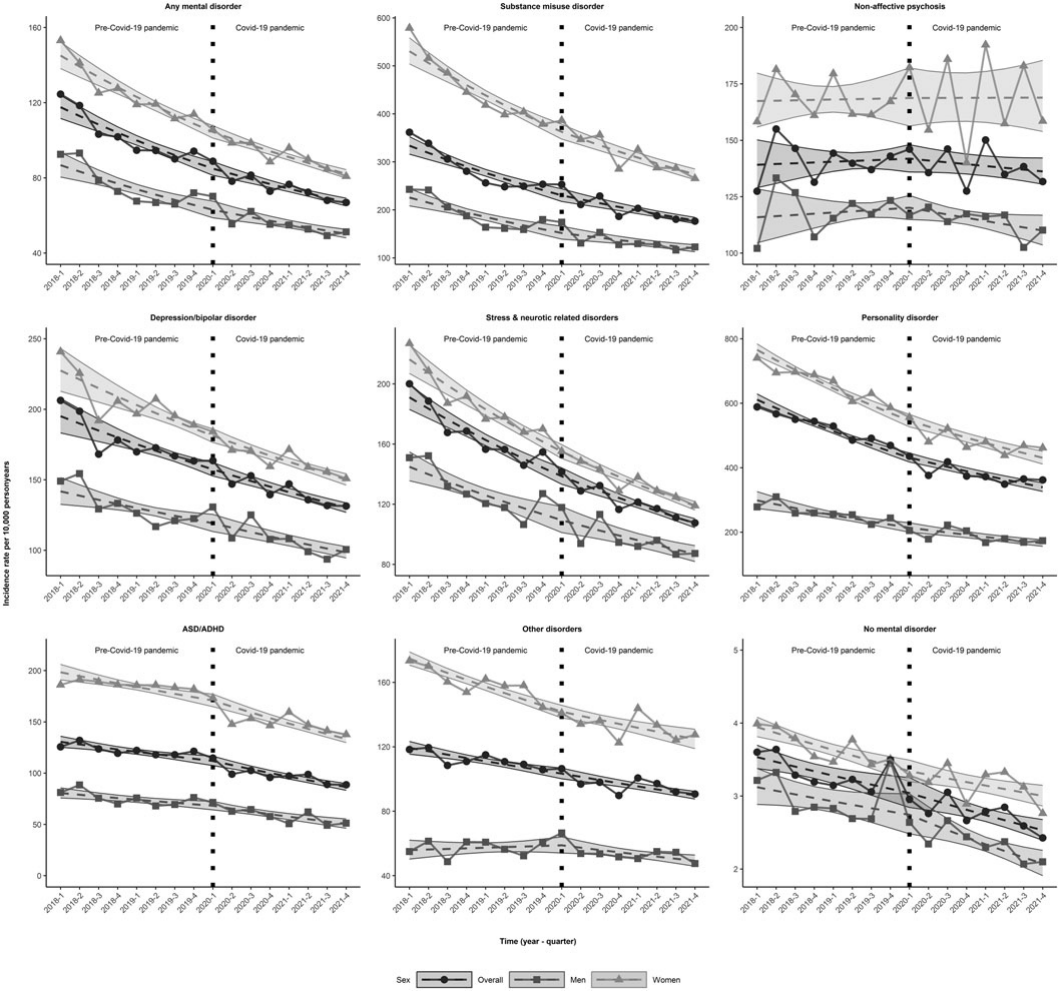
Crude observed and estimated incidence rates of suicide attempt per 10 000 person-years with 95% confidence intervals during the years preceding the start of the Covid-19 pandemic (first quarter 2018 or 2018Q1–2020Q1) and the years after start of the pandemic (2020Q2–2021Q4) for individuals with specific severe mental disorders treated in specialized healthcare and individuals without such treated disorders the year preceding the start of the respective quarter, covering the entire population > 10 years of age in Sweden, stratified by sex; ASD/ADHD, Autism spectrum disorders/Attention deficit hyperactivity disorders.

The IRs (95% CIs) per 10 000 person-years for suicide attempt in individuals with any pre-existing SMD decreased from 117.6 (111.6–123.9) in 2018Q1 to 84.9 (81.4–88.6) in the quarter before the start of the pandemic (2020Q1) and continued to decline to 67.1 (65.1–69.2) in 2020Q4 (Fig. 2 and online Supplementary Table S3). For specific SMDs, suicide attempt rates in 2018Q1 were highest among individuals with personality disorder (610.9; 593.5–628.9) and substance use disorder (333.8; 316.0–352.5). Similar sex-specific rates were observed in 2018Q1. Women with personality disorder had the highest IRs for suicide attempt (765.6; 747.4–784.2) across all sex and SMD diagnosis specific groups.

Both before and during the pandemic, IRs of suicide attempt either declined slightly or remained stable for all diagnostic groups of pre-existing SMDs (Table 3). The relative change in suicide attempt rates during *v.* pre-pandemic (ratio of change in IR, 95% CIs 1.007, 0.99–1.02) was also stable for all diagnostic groups. These findings were similar for individuals without a pre-existing SMD (Table 3).

**Table 3.** Change in crude Incidence Rates (IR)^a^ before (first quarter 2018 or 2018Q1–2020Q1) and during the Covid-19 pandemic (2020Q2 –2021Q4) and the ratio of change in IRs during *v.* before the pandemic^b^ of suicide attempt for groups of individuals with/without pre-existing severe mental disorders treated in specialised healthcare the year preceding the respective quarter

	Pre-Covid-19 pandemic		Covid-19 pandemic		During v. before Covid-19 pandemic	
Mental health disorder	Change in IR (95% CI)	*p* value	Change in IR (95% CI)	*p* value	Ratio of change in IR (95% CI)	*p* value
Any mental disorders	0.96 (0.95–0.97)	<0.001	0.97 (0.96–0.97)	<0.001	1.011 (0.99–1.02)	0.411
Substance misuse disorder	0.95 (0.94–0.97)	<0.001	0.96 (0.95–0.97)	<0.001	1.01 (0.99–1.03)	0.350
Non-affective psychosis	1.00 (0.99–1.02)	0.725	0.99 (0.99–1.00)	0.167	0.99 (0.97–1.01)	0.419
Depression/Bipolar disorder	0.97 (0.96–0.98)	<0.001	0.97 (0.97–0.98)	<0.001	1.00 (0.98–1.01)	0.973
Stress & neurotic related disorders	0.96 (0.95–0.97)	<0.001	0.96 (0.96–0.97)	<0.001	1.00 (0.99–1.02)	0.642
Personality disorder	0.96 (0.95–0.96)	<0.001	0.97 (0.96–0.97)	<0.001	1.01 (0.99–1.02)	0.130
ASD/ADHD^c^	0.98 (0.97–0.99)	<0.001	0.96 (0.95–0.97)	<0.001	0.98 (0.97–0.99)	0.039
Other mental disorders	0.98 (0.97–0.99)	<0.001	0.98 (0.97–0.99)	<0.001	0.99 (0.99–1.01)	0.748
No mental disorders	0.98 (0.97–0.99)	0.002	0.97 (0.96–0.99)	0.001	0.99 (0.97–1.02)	0.559

a Slopes based on the GEE models with a linear fit showing the % change in IR for one unit (quarter) change during the pre- and pandemic periods.

b Estimates of slopes during the pandemic divided by the estimates of slopes before the pandemic.

c Autism spectrum disorders/ Attention Deficit Hyperactivity Disorders.

### Sensitivity analyses

The results from the sensitivity analyses adjusting for the covariates showed similar patterns (online Supplementary Figs S1 and S2 and Tables S2 and S4) to our main analysis for both suicide and suicide attempt. The sensitivity analysis (1) excluding events of undetermined intent as suicide/suicide attempt and (2) excluding individuals older than 66 years revealed similar patterns (data not shown) to the results from the main analyses.

## Discussion

### Main findings

This study comprising the entire population of Sweden above 10 years of age, with an observation window of 3.5 to 4 years, showed that suicide rates in individuals with pre-existing SMDs declined during the pandemic, both in absolute terms and in relation to the pre-pandemic period. In contrast, suicide attempt showed stable trends for individuals with pre-existing SMDs (comparing the pandemic period to the pre-pandemic period). These discrepancies were most evident for individuals with substance misuse disorder and ASD/ADHD where percentage changes (decreases) in IRs of suicide *v.* suicide attempt for one quarter during the pandemic were the following: substance misuse (11.2% *v.* 3.6%, respectively) and ASD/ADHD (10.7% *v.* 3.6%). The patterns for individuals without pre-existing SMDs were similar for IRs of suicide and suicide attempt, namely stable during as compared to before the pandemic. No sex differences in the associations were found.

Our observed decreases in suicide rates in individuals with pre-existing SMDs were stronger than those for individuals in the general population reported earlier (Pirkis et al., 2021, 2022). Potential explanations for these paradoxical findings may include reduced access to means and a rapid and positive pandemic response of the social insurance and healthcare services (Pirkis et al., 2021, 2022). The fact that Sweden applied modestly strict mitigation measures with a considerable focus on mental health might have contributed to these positive findings. Particularly reduced access to means might be an explanation behind the strong decreases in the IRs of suicide in individuals with substance misuse disorder during the pandemic. Lower availability of illicit drugs due to closing routes of their import and consequent higher street prices may have contributed to decreasing trends of suicide during the pandemic (Farhoudian et al., 2021; Lindqvist, Wallmofeldt, Holmen, Hammarberg, & Kaberg, 2021).

Decreases in suicide rates in individuals with pre-existing SMDs are in contrast to the findings on trends for suicide attempt showing primarily stable trends. The few available studies examining the risk of suicide attempt in the general population (Dube et al., 2021) and in individuals with specific mental disorders (Berardelli et al., 2021; Li et al., 2022) showed increases in these groups. Differences in findings with our study might be related to the fact that the latter studies were conducted in other countries with different healthcare services, social insurance measures as well as with more restrictive public health measures during the pandemic. The discrepancy in findings between decreases in suicide rates and the stable suicide attempt rates for individuals with pre-existing SMDs is thought-provoking and warrants further studies. Apart from the potential underreporting of suicide attempts in specialized healthcare, these findings might be due to the fact that younger individuals and women were shown to be more vulnerable for mental ill-health during the pandemic and these are also the groups with higher risks for suicide attempt (Kunzler et al., 2021; Manchia et al., 2022).

### Strengths and limitations

The primary strength of this study is the use of an advanced analytical strategy accounting for pre-pandemic suicidal behavior trends and providing information on the in-depth and long-term consequences of the Covid-19 pandemic on rates of suicidal behavior in groups of individuals with specific pre-existing SMDs. The use of high-quality register data covering the entire population in Sweden limits the possibility of selection bias from non-response and loss to follow-up. Moreover, studying rare outcomes such as suicide among vulnerable but relatively small risk groups is another strength of this study which is, otherwise, quite challenging without big data.

Some limitations should also be mentioned. First, suicide attempts and pre-existing SMDs were measured by specialized healthcare data, limiting the generalizability to less severe forms of morbidity. An underestimation of ‘true’ suicide attempt rates is, therefore, expected in this study. Additionally, the group without pre-existing SMD during the year prior to cohort entry may include individuals who had a history of mental disorder before this inclusion period and therefore, we may have overestimated the rates of suicidal behavior in this group. This overestimation might, though, be negligible due to the size of the general population (including several million individuals). The use of information on specialized health care can also be interpreted as a strength because these registered events are physician-diagnosed and considered to be more objective than self-reports. Moreover, the aim of this study was to investigate rates of suicidal behavior in individuals with SMDs and mental disorders treated in specialized healthcare can be regarded as of high medical severity. With regard to the statistics on suicide deaths, it is likely that some case ascertainment is prolonged and a number of deaths due to suicide would have been added retrospectively if we had had data for the entire year 2021. This is, however, not a likely explanation for the observed trends in suicide rates during the pandemic as such not-yet ascertained cases would be few. We analyzed trends for suicide attempt up until 2021Q4 and for suicide, somewhat shorter, i.e. until 2021Q2. It is theoretically possible that the model for suicide could have been affected by exceptionally high rates in the third and fourth quarter of 2021 (data which was not yet available). This is, however, unlikely as the national statistics published by the National Centre for Suicide Research and Prevention, Sweden show comparable rates for suicide in 2020 and 2021. Furthermore, we could not adjust for behavioral factors e.g. alcohol consumption, due to the lack of such data in the registers. Finally, findings are not generalizable to countries with healthcare and social insurance systems that differ significantly from those in Sweden.

## Conclusions

This study revealed that compared to prior to the pandemic, suicide rates decreased during the pandemic, while rates for suicide attempt showed stable trends for individuals with pre-existing SMDs. As the decrease in suicide during the pandemic for individuals with pre-existing SMDs was quite pronounced, further studies aiming to understand possible mechanisms behind these trends are highly warranted to consult future suicide prevention programs.

## Supporting information

Mittendorfer-Rutz et al. supplementary material Mittendorfer-Rutz et al. supplementary material

## Data Availability

The data used in this study cannot be made publicly available due to privacy regulations. According to the General Data Protection Regulation, the Swedish law SFS 2018:218, the Swedish Data Protection Act, the Swedish Ethical Review Act, and the Public Access to Information and Secrecy Act, these types of sensitive data can only be made available for specific purposes, including research, that meets the criteria for access to this sort of sensitive and confidential data as determined by a legal review. Readers may contact Professor Kristina Alexanderson (kristina.alexanderson@ki.se) regarding the data.

## Supplementary material

The supplementary material for this article can be found at https://doi.org/10.1017/S003329172400299X
