# Supplementary material for: Suicidal behavior in patients with severe mental disorders prior to and during the COVID-19 pandemic
Source: Psychol Med. 2024 Dec 16;54(16):4759–67. doi: 10.1017/S003329172400299X (PMC11769905; doi:10.1017/S003329172400299X)
Supplement: Mittendorfer-Rutz et al. supplementary material [file S003329172400299Xsup001.docx]

**Supplementary Table 1.** Incidence rates of suicide per 10,000 person-years with 95% confidence intervals during the years preceding the start of the Covid-19 pandemic (first quarter 2018 or 2018Q1 – 2020Q1) and the years after start of the pandemic (2020Q2 - 2021Q2) for individuals with specific severe mental disorders treated in specialised healthcare and individuals without such treated disorders the year preceding the start of the respective quarter, covering the entire population > 10 years of age in Sweden, stratified by sex; ASD/ADHD - Autism spectrum disorders/Attention deficit hyperactivity disorders.

|  |  | 2018Q1 | 2018Q2 | 2018Q3 | 2018Q4 | 2019Q1 | 2019Q2 | 2019Q3 | 2019Q4 | 2020Q1 | 2020Q2 | 2020Q3 | 2020Q4 | 2021Q1 | 2021Q2 |
| --- | --- | --- | --- | --- | --- | --- | --- | --- | --- | --- | --- | --- | --- | --- | --- |
| Any mental disorder | All | 16.0 (15.0-17.1) | 15.4 (14.5-16.3) | 14.8 (14.0-15.5) | 14.2 (13.5-14.9) | 13.6 (13.0-14.3) | 13.1 (12.5-13.8) | 12.6 (11.9-13.3) | 12.1 (11.3-12.9) | 11.6 (10.8-12.5) | 10.4 (9.9-11.0) | 9.3 (9.0-9.7) | 8.4 (8.1-8.7) | 7.5 (7.1-7.9) | 6.7 (6.3-7.2) |
|  | Men | 20.9 (19.3-22.7) | 20.1 (18.8-21.5) | 19.4 (18.3-20.5) | 18.6 (17.7-19.6) | 17.9 (17.1-18.8) | 17.2 (16.4-18.2) | 16.6 (15.6-17.6) | 16.0 (14.9-17.1) | 15.4 (14.1-16.7) | 13.8 (13.0-14.7) | 12.4 (11.9-12.9) | 11.1 (10.8-11.5) | 10.0 (9.6-10.4) | 9.0 (8.5-9.5) |
|  | Women | 11.6 (10.9-12.4) | 11.1 (10.5-11.8) | 10.6 (10.1-11.3) | 10.2 (9.7-10.8) | 9.8 (9.2-10.4) | 9.4 (8.8-10.0) | 9.0 (8.4-9.7) | 8.6 (7.9-9.3) | 8.3 (7.5-9.0) | 7.4 (6.9-7.9) | 6.6 (6.2-7.0) | 5.9 (5.5-6.3) | 5.3 (4.8-5.8) | 4.7 (4.2-5.4) |
| Sustance misuse disorder | All | 42.6 (38.2-47.5) | 40.9 (37.1-45.0) | 39.2 (35.9-42.7) | 37.6 (34.7-40.7) | 36.0 (33.3-38.9) | 34.5 (31.9-37.4) | 33.1 (30.4-36.0) | 31.7 (28.9-34.8) | 30.4 (27.4-33.8) | 27.0 (25.1-29.1) | 24.0 (22.8-25.2) | 21.3 (20.3-22.4) | 18.9 (17.6-20.3) | 16.8 (15.2-18.6) |
|  | Men | 43.4 (39.5-47.7) | 41.6 (38.3-45.3) | 40.0 (37.1-43.1) | 38.3 (35.8-41.1) | 36.8 (34.5-39.2) | 35.3 (33.1-37.6) | 33.9 (31.7-36.2) | 32.5 (30.3-34.9) | 31.2 (28.8-33.7) | 28.1 (26.5-29.7) | 25.2 (23.8-26.7) | 22.7 (21.0-24.5) | 20.4 (18.4-22.7) | 18.4 (16.0-21.1) |
|  | Women | 40.9 (34.2-48.9) | 39.2 (33.6-45.7) | 37.6 (32.9-42.9) | 36.0 (32.0-40.5) | 34.5 (30.8-38.6) | 33.0 (29.4-37.1) | 31.7 (27.8-36.1) | 30.3 (26.1-35.2) | 29.1 (24.4-34.6) | 25.2 (22.1-28.6) | 21.8 (19.7-24.2) | 18.9 (16.9-21.2) | 16.4 (14.1-19.1) | 14.2 (11.6-17.4) |
| Non-affective psychosis | All | 36.9 (30.4-44.8) | 35.3 (29.9-41.6) | 33.6 (29.3-38.6) | 32.1 (28.6-36.0) | 30.6 (27.8-33.7) | 29.2 (26.8-32.0) | 27.9 (25.4-30.6) | 26.6 (23.9-29.7) | 25.4 (22.3-28.9) | 23.8 (21.6-26.1) | 22.2 (20.4-24.1) | 20.7 (18.8-22.9) | 19.4 (16.9-22.2) | 18.1 (15.1-21.7) |
|  | Men | 51.0 (44.5-58.4) | 47.7 (42.5-53.6) | 44.6 (40.3-49.4) | 41.7 (37.9-45.9) | 39.1 (35.4-43.1) | 36.5 (32.7-40.8) | 34.2 (30.1-38.8) | 32.0 (27.6-37.1) | 29.9 (25.2-35.6) | 29.2 (25.8-33.1) | 28.5 (26.0-31.2) | 27.8 (25.4-30.4) | 27.1 (24.0-30.6) | 26.5 (22.3-31.4) |
|  | Women | 18.3 (10.7-31.2) | 18.5 (11.8-29.0) | 18.7 (12.9-26.9) | 18.8 (14.2-25.1) | 19.0 (15.4-23.5) | 19.2 (16.6-22.3) | 19.4 (17.2-22.0) | 19.6 (16.8-22.9) | 19.8 (15.9-24.7) | 16.7 (14.1-19.9) | 14.1 (11.7-17.0) | 11.9 (9.2-15.3) | 10.0 (7.1-14.1) | 8.4 (5.4-13.2) |
| Depression/bipolar disorder | All | 24.5 (22.5-26.6) | 23.7 (22.1-25.5) | 23.0 (21.6-24.5) | 22.3 (21.0-23.5) | 21.6 (20.4-22.7) | 20.9 (19.8-22.1) | 20.2 (19.1-21.5) | 19.6 (18.3-21.0) | 19.0 (17.6-20.5) | 17.1 (16.2-18.1) | 15.4 (14.7-16.1) | 13.8 (13.2-14.6) | 12.5 (11.6-13.4) | 11.2 (10.2-12.4) |
|  | Men | 37.0 (33.2-41.2) | 35.6 (32.5-39.0) | 34.3 (31.8-36.9) | 33.0 (30.9-35.2) | 31.8 (29.8-33.8) | 30.6 (28.6-32.7) | 29.4 (27.2-31.9) | 28.3 (25.7-31.2) | 27.3 (24.3-30.6) | 25.0 (22.9-27.2) | 22.8 (21.6-24.2) | 20.9 (20.2-21.6) | 19.1 (18.5-19.8) | 17.5 (16.5-18.5) |
|  | Women | 16.9 (15.0-19.1) | 16.5 (14.8-18.3) | 16.1 (14.7-17.6) | 15.7 (14.4-17.0) | 15.3 (14.2-16.4) | 14.9 (13.8-16.0) | 14.5 (13.4-15.7) | 14.1 (13.0-15.4) | 13.8 (12.5-15.2) | 12.1 (11.3-13.0) | 10.7 (9.9-11.4) | 9.4 (8.5-10.3) | 8.2 (7.2-9.4) | 7.2 (6.1-8.7) |
| Stress and neurotic related disorder | All | 21.3 (19.3-23.6) | 20.4 (18.7-22.2) | 19.5 (18.1-21.0) | 18.7 (17.4-20.0) | 17.9 (16.6-19.2) | 17.1 (15.7-18.5) | 16.3 (14.8-18.0) | 15.6 (14.0-17.5) | 14.9 (13.1-17.1) | 13.6 (12.3-15.0) | 12.3 (11.5-13.2) | 11.2 (10.5-11.8) | 10.1 (9.4-10.9) | 9.2 (8.3-10.2) |
|  | Men | 32.7 (28.8-37.1) | 31.3 (28.2-34.8) | 30.0 (27.4-32.9) | 28.7 (26.4-31.3) | 27.5 (25.1-30.1) | 26.3 (23.7-29.2) | 25.2 (22.3-28.5) | 24.1 (20.9-27.9) | 23.1 (19.5-27.4) | 21.1 (18.6-24.0) | 19.3 (17.7-21.1) | 17.6 (16.6-18.8) | 16.1 (15-17.3) | 14.7 (13.2-16.4) |
|  | Women | 15.2 (13.4-17.1) | 14.4 (13.0-16.0) | 13.8 (12.6-15.0) | 13.1 (12.1-14.2) | 12.5 (11.6-13.5) | 11.9 (11.0-12.9) | 11.4 (10.4-12.4) | 10.9 (9.8-12.0) | 10.3 (9.2-11.7) | 9.3 (8.5-10.2) | 8.3 (7.7-9.0) | 7.5 (6.9-8.2) | 6.7 (6.1-7.5) | 6.0 (5.3-6.9) |
| Personality Disorder | All | 35.4 (31.8-39.5) | 34.8 (31.5-38.4) | 34.2 (31.1-37.6) | 33.6 (30.6-36.9) | 33.0 (29.9-36.5) | 32.5 (29.2-36.1) | 31.9 (28.3-35.9) | 31.3 (27.4-35.8) | 30.8 (26.5-35.7) | 27.7 (24.9-30.7) | 24.8 (22.8-27.1) | 22.3 (20.0-24.9) | 20.0 (17.1-23.4) | 18.0 (14.5-22.3) |
|  | Men | 39.6 (28.7-54.6) | 38.3 (29.5-49.7) | 37.0 (30.1-45.4) | 35.7 (30.4-42.0) | 34.5 (30.0-39.8) | 33.3 (28.6-38.8) | 32.2 (26.6-39.0) | 31.1 (24.4-39.7) | 30.1 (22.3-40.6) | 28.6 (22.6-36.1) | 27.1 (22.7-32.3) | 25.7 (22.5-29.4) | 24.4 (21.5-27.8) | 23.2 (19.7-27.3) |
|  | Women | 33.2 (28.6-38.4) | 32.9 (28.7-37.8) | 32.7 (28.6-37.3) | 32.4 (28.4-37.0) | 32.2 (28.0-37.0) | 31.9 (27.5-37.0) | 31.7 (26.9-37.3) | 31.4 (26.3-37.6) | 31.2 (25.6-38.0) | 26.9 (23.5-30.8) | 23.2 (20.2-26.7) | 20.0 (16.3-24.5) | 17.3 (12.9-23.1) | 14.9 (10.1-21.9) |
| ASD/ADHD | All | 11.7 (10.7-12.7) | 11.4 (10.6-12.2) | 11.1 (10.5-11.7) | 10.8 (10.2-11.3) | 10.5 (9.9-11.1) | 10.2 (9.5-11.0) | 10.0 (9.1-10.9) | 9.7 (8.7-10.9) | 9.5 (8.3-10.8) | 8.4 (7.6-9.4) | 7.5 (6.8-8.4) | 6.7 (5.9-7.7) | 6.0 (5.1-7.1) | 5.4 (4.3-6.7) |
|  | Men | 12.6 (11.3-14.0) | 12.2 (11.3-13.1) | 11.8 (11.2-12.4) | 11.4 (11.0-11.8) | 11.0 (10.5-11.5) | 10.6 (9.9-11.4) | 10.3 (9.3-11.4) | 10.0 (8.7-11.4) | 9.6 (8.2-11.3) | 8.9 (7.8-10.2) | 8.2 (7.2-9.4) | 7.6 (6.5-9.0) | 7.0 (5.7-8.7) | 6.5 (5.0-8.5) |
|  | Women | 10.3 (8.6-12.4) | 10.2 (8.8-11.9) | 10.1 (8.8-11.4) | 9.9 (8.8-11.1) | 9.8 (8.7-11.0) | 9.6 (8.5-10.9) | 9.5 (8.2-11.0) | 9.4 (7.9-11.2) | 9.3 (7.6-11.3) | 7.7 (6.6-9.0) | 6.4 (5.5-7.5) | 5.4 (4.4-6.6) | 4.5 (3.4-5.9) | 3.7 (2.6-5.4) |
| Other disorder | All | 8.6 (7.3-10.3) | 8.7 (7.7-9.9) | 8.8 (8.1-9.6) | 8.9 (8.3-9.5) | 9.0 (8.3-9.7) | 9.0 (8.0-10.2) | 9.1 (7.7-10.7) | 9.2 (7.4-11.4) | 9.3 (7.1-12.0) | 8.3 (6.8-10.2) | 7.5 (6.4-8.7) | 6.7 (6.0-7.5) | 6.0 (5.5-6.7) | 5.4 (4.8-6.1) |
|  | Men | 12.4 (9.8-15.7) | 12.2 (10.1-14.7) | 11.9 (10.3-13.8) | 11.7 (10.5-13.1) | 11.5 (10.3-12.8) | 11.3 (9.9-12.9) | 11.1 (9.4-13.2) | 10.9 (8.8-13.6) | 10.7 (8.2-14.0) | 9.9 (8.0-12.2) | 9.2 (7.8-10.7) | 8.5 (7.5-9.6) | 7.8 (7.0-8.7) | 7.2 (6.3-8.3) |
|  | Women | 5.3 (3.6-7.9) | 5.6 (4.0-7.8) | 5.9 (4.5-7.7) | 6.2 (5.0-7.7) | 6.5 (5.5-7.7) | 6.9 (5.9-8.0) | 7.3 (6.2-8.5) | 7.7 (6.3-9.4) | 8.1 (6.3-10.4) | 6.9 (5.7-8.4) | 6.0 (5.2-6.9) | 5.1 (4.6-5.8) | 4.4 (3.9-5.1) | 3.8 (3.2-4.6) |
| No mental disorder | All | 1.0 (0.9-1.1) | 1.0 (0.9-1.0) | 0.9 (0.9-1.0) | 0.9 (0.9-1.0) | 0.9 (0.9-0.9) | 0.9 (0.9-0.9) | 0.9 (0.8-0.9) | 0.9 (0.8-0.9) | 0.8 (0.8-0.9) | 0.8 (0.8-0.9) | 0.8 (0.8-0.8) | 0.8 (0.8-0.8) | 0.8 (0.7-0.8) | 0.8 (0.7-0.8) |
|  | Men | 1.5 (1.4-1.6) | 1.4 (1.4-1.5) | 1.4 (1.4-1.5) | 1.4 (1.3-1.4) | 1.4 (1.3-1.4) | 1.3 (1.3-1.4) | 1.3 (1.3-1.4) | 1.3 (1.2-1.4) | 1.3 (1.2-1.3) | 1.3 (1.2-1.3) | 1.2 (1.2-1.3) | 1.2 (1.2-1.3) | 1.2 (1.1-1.3) | 1.2 (1.1-1.3) |
|  | Women | 0.5 (0.4-0.6) | 0.5 (0.4-0.6) | 0.5 (0.4-0.5) | 0.5 (0.4-0.5) | 0.4 (0.4-0.5) | 0.4 (0.4-0.5) | 0.4 (0.4-0.5) | 0.4 (0.4-0.4) | 0.4 (0.3-0.4) | 0.4 (0.3-0.4) | 0.3 (0.3-0.4) | 0.3 (0.3-0.4) | 0.3 (0.3-0.3) | 0.3 (0.3-0.3) |

**Supplementary Figure 1:** Multivariate-adjusted* incidence rates with 95% confidence intervals of suicide per 10,000 person-years during the years preceding the start of the Covid-19 pandemic (first quarter 2018 or 2018Q1 - 2020Q1) and the years after start of the pandemic (2020Q2 - 2021Q2) for individuals with specific severe mental disorders treated in specialised healthcare and individuals without such treated disorders the year preceding the start of the respective quarter, covering the entire population > 10 years of age in Sweden

*Adjusted for sex, age, educational level, family situation, country of birth, type of living area, previous sickness absence, disability pension and/or unemployment; ASD/ADHD - Autism spectrum disorders/Attention Deficit Hyperactivity Disorder

Submitted as separate file

**Mathematic basis for the model used in the analyses**

The general form of the estimated models was:

$$log\left( n_{q} \right)=\alpha+ \beta_{1}\times{T1}_{q}+\beta_{2}\times{T2}_{q}+S_{q}+P_{q}+interactions$$

where *n_q_* is the number of cases (death by suicide or suicide attempt) at quarter *q* from study start. *T1_q_* is the time as a continuous variable ranging from 1 to14 for death by suicide and 1 to 16 for suicide attempt, where *β_1_* is an estimate of the pre-Covid-19 IR. *T2_q_* is an additional time variable which permits the Covid-19 pandemic IR, estimated by *T1*, to shift at quarter 9, i.e. the start of the pandemic. This is accomplished by setting *T2* to 0 pre-Covid-19 pandemic and *T1_q_*-14 (or 16) Covid-19 pandemic. *β_2_* is the estimated difference in IRs between pre-Covid-19 and Covid-19 pandemic. The IR for the Covid-19 pandemic is the sum of *β_1_* and *β_2_*. *S_q_* are seasonal variables parametrised as Fourier terms (Bhaskaran K et al., 2013) consisting of a pair of sine and cosine functions capturing the time periodicity. The Fourier expression was used when analysing death by suicide and was expressed as *cosine (2×π ×q)/4* + *sinus (π×q)/4*. *P_q_* is an offset variable, *log(total number of days observed in q),* used to account for variation in population size of each quarter.

As ITS is a quasi-experimental design (within-individual analysis) there is no need to control for confounding. However, the IRs can differ between subsets of the study population. To evaluate different IRs between the *i* sexes (man, women), interaction terms was added to the model as *sex_i_×T1_q_*, *sex_i_×T2_q_* and *sex_i_×S_q_*. The model with sex as an effect modifier also included sex as a main effect, *sex_i_* and all the added model parameters permitted the IRs and seasonal effects to differ between sexes.

**Supplementary Table 2:** Multivariate-adjusted^1^ change in Incidence Rates (IR)^2^ before (first quarter 2018 or 2018Q1 – 2020Q1) and during the Covid-19 pandemic (2020Q2 –2021Q2) and the ratio of change in IRs during versus before the pandemic^3^ of suicide for groups of individuals with/without pre-existing severe mental disorders treated in specialised healthcare the year preceding the respective quarter.

|  | **Before Covid-19 pandemic** | | **During Covid-19 pandemic** | | **During vs before Covid-19 pandemic** | |
| --- | --- | --- | --- | --- | --- | --- |
| **Mental disorder** | **Change in IR (95% CI)** | **P-value** | **Change in IR (95% CI)** | **P-value** | **Ratio of change in IR (95% CI)** | **P-value** |
| Substance misuse | 0.97 (0.95-0.99) | 0.012 | 0.89 (0.85-0.93) | <0.001 | 0.92 (0.86-0.97) | 0.005 |
| Non-affective psychosis | 0.96 (0.92-1.00) | 0.048 | 0.94 (0.87-1.01) | 0.091 | 0.98 (0.88-1.08) | 0.652 |
| Depression/Bipolar disorder | 0.98 (0.95-1.00) | 0.055 | 0.90 (0.86-0.94) | <0.001 | 0.92 (0.87-0.98) | 0.007 |
| Stress & neurotic-rel. disorders | 0.96 (0.94-0.99) | 0.003 | 0.91 (0.87-0.95) | <0.001 | 0.94 (0.89-0.99) | 0.035 |
| Personality disorder | 0.99 (0.94-1.03) | 0.624 | 0.89 (0.83-0.97) | 0.009 | 0.91 (0.81-1.02) | 0.098 |
| Other mental disorders | 1.01 (0.97-1.05) | 0.628 | 0.90 (0.84-0.97) | 0.004 | 0.89 (0.80-0.99) | 0.026 |
| ASD/ADHD^4^ | 0.98 (0.94-1.02) | 0.231 | 0.89 (0.84-0.95) | 0.001 | 0.91 (0.83-1.00) | 0.052 |
| No mental disorders | 0.98 (0.96-0.99) | 0.041 | 0.98 (0.94-1.01) | 0.196 | 0.99 (0.95-1.04) | 0.867 |

^1^Adjusted for sex, age, educational level, family situation, country of birth, type of living area, previous sickness absence, disability pension and/or unemployment
^2^Slopes based on the GEE models with a linear fit showing the % change in IR for one unit (quarter) change during the pre- and pandemic periods.
^3^Estimates of slopes during the pandemic divided by the estimates of slopes before the pandemic.
^4^Autism spectrum disorders/Attention deficit hyperactivity disorders.

**Supplementary Table 3.** Incidence rates of suicide attempt per 10,000 person-years with 95% confidence intervals during the years preceding (first quarter 2018 or 2018Q1 – 2020Q1) and following the COVID-19 pandemic (2020Q2 - 2021Q4) for individuals with specific severe mental disorders treated in specialised healthcare and individuals without such treated disorders the year preceding the start of the respective quarter, covering the entire population > 10 years of age in Sweden, stratified by sex; ASD/ADHD - Autism spectrum disorders/Attention deficit hyperactivity disorders.

|  |  | 2018Q1 | 2018Q2 | 2018Q3 | 2018Q4 | 2019Q1 | 2019Q2 | 2019Q3 | 2019Q4 | 2020Q1 | 2020Q2 | 2020Q3 | 2020Q4 | 2021Q1 | 2021Q2 | 2021Q3 | 2021Q4 |
| --- | --- | --- | --- | --- | --- | --- | --- | --- | --- | --- | --- | --- | --- | --- | --- | --- | --- |
| Any mental disorder | All | 117.6 (111.6-123.9) | 112.9 (108.1-117.9) | 108.4 (104.6-112.3) | 104.1 (101.1-107.2) | 99.9 (97.4-102.5) | 95.9 (93.6-98.4) | 92.1 (89.5-94.8) | 88.4 (85.4-91.6) | 84.9 (81.4-88.6) | 82.1 (79.2-85.1) | 79.4 (77.1-81.8) | 76.8 (74.9-78.7) | 74.2 (72.6-75.9) | 71.8 (70.2-73.4) | 69.4 (67.7-71.2) | 67.1 (65.1-69.2) |
|  | Men | 86.8 (80.5-93.6) | 83.4 (78.4-88.7) | 80.1 (76.3-84.1) | 77.0 (74.0-80.0) | 73.9 (71.4-76.5) | 71.0 (68.5-73.7) | 68.3 (65.3-71.4) | 65.6 (62.0-69.4) | 63.0 (58.8-67.6) | 61.0 (57.5-64.7) | 59.1 (56.2-62.1) | 57.2 (54.8-59.7) | 55.4 (53.4-57.5) | 53.6 (51.7-55.6) | 51.9 (49.9-54.0) | 50.3 (48.0-52.6) |
|  | Women | 145.1 (138.1-152.3) | 139.2 (133.6-145.1) | 133.6 (129.1-138.3) | 128.2 (124.7-131.9) | 123.1 (120.2-126.0) | 118.1 (115.6-120.6) | 113.4 (110.9-115.9) | 108.8 (106.0-111.7) | 104.4 (101.2-107.8) | 100.9 (98.3-103.6) | 97.5 (95.4-99.6) | 94.2 (92.5-96.0) | 91.0 (89.4-92.6) | 88.0 (86.3-89.6) | 85.0 (83.1-86.9) | 82.1 (79.9-84.4) |
| Sustance misuse disorder | All | 333.8 (316.0-352.5) | 318.6 (305.0-332.9) | 304.2 (293.9-314.8) | 290.4 (282.6-298.5) | 277.2 (270.4-284.3) | 264.7 (257.2-272.4) | 252.7 (243.7-262) | 241.2 (230.3-252.6) | 230.3 (217.5-243.8) | 222.0 (211.2-233.3) | 213.9 (204.9-223.4) | 206.2 (198.5-214.1) | 198.7 (192.1-205.6) | 191.5 (185.4-197.8) | 184.6 (178.6-190.8) | 177.9 (171.6-184.5) |
|  | Men | 225.7 (208.4-244.4) | 214.8 (201.5-228.9) | 204.4 (194.5-214.9) | 194.5 (186.9-202.5) | 185.1 (178.2-192.3) | 176.2 (168.5-184.2) | 167.7 (158.4-177.5) | 159.6 (148.5-171.5) | 151.9 (139.0-165.9) | 146.8 (136.1-158.3) | 141.9 (133.1-151.3) | 137.2 (129.9-144.9) | 132.6 (126.3-139.3) | 128.2 (122.2-134.6) | 123.9 (117.6-130.6) | 119.8 (112.7-127.3) |
|  | Women | 530.6 (504.1-558.4) | 505.9 (485.0-527.7) | 482.4 (466.3-499.1) | 460.0 (447.7-472.6) | 438.6 (428.7-448.8) | 418.2 (408.7-428.0) | 398.8 (388.1-409.8) | 380.3 (367.5-393.4) | 362.6 (347.6-378.3) | 348.3 (335.4-361.7) | 334.6 (323.3-346.3) | 321.5 (311.3-332.0) | 308.8 (299.1-318.8) | 296.7 (286.9-306.7) | 285.0 (274.8-295.6) | 273.8 (262.9-285.1) |
| Non-affective psychosis | All | 139.2 (128.9-150.2) | 139.5 (130.9-148.7) | 139.8 (132.8-147.2) | 140.2 (134.6-146.0) | 140.5 (136.2-144.9) | 140.8 (137.3-144.4) | 141.2 (137.5-144.9) | 141.5 (137.0-146.2) | 141.8 (135.9-148.0) | 141.0 (135.8-146.4) | 140.2 (135.6-145.0) | 139.4 (135.1-143.9) | 138.6 (134.3-143.1) | 137.8 (133.2-142.5) | 137.0 (132.0-142.2) | 136.2 (130.5-142.1) |
|  | Men | 115.9 (104.5-128.4) | 116.4 (106.7-126.9) | 116.9 (108.9-125.5) | 117.4 (111.1-124.2) | 118.0 (113.1-123.0) | 118.5 (114.9-122.2) | 119.0 (115.8-122.3) | 119.5 (115.7-123.5) | 120.1 (114.9-125.5) | 118.6 (114.5-122.8) | 117.1 (113.9-120.4) | 115.6 (112.7-118.7) | 114.2 (110.8-117.7) | 112.8 (108.6-117.2) | 111.4 (106.1-116.9) | 110.0 (103.6-116.8) |
|  | Women | 167.4 (155.9-179.8) | 167.6 (158.2-177.5) | 167.7 (160.3-175.5) | 167.9 (161.9-174.1) | 168.1 (162.5-173.8) | 168.2 (161.8-174.9) | 168.4 (160.4-176.8) | 168.6 (158.5-179.3) | 168.7 (156.4-182.1) | 168.8 (157.6-180.7) | 168.8 (158.3-180.0) | 168.8 (158.4-179.9) | 168.8 (157.9-180.5) | 168.9 (156.9-181.7) | 168.9 (155.6-183.4) | 168.9 (153.9-185.4) |
| Depression/bipolar disorder | All | 195.1 (183.3-207.6) | 189.9 (180.2-200.1) | 184.9 (177.1-193.0) | 180.0 (174.0-186.2) | 175.2 (170.7-179.8) | 170.6 (167.2-174.1) | 166.1 (162.9-169.3) | 161.7 (158.0-165.5) | 157.4 (152.7-162.3) | 153.2 (149.2-157.3) | 149.1 (145.7-152.5) | 145.1 (142.2-148.1) | 141.2 (138.5-144.0) | 137.5 (134.7-140.3) | 133.8 (130.8-136.8) | 130.2 (126.9-133.6) |
|  | Men | 141.7 (132.6-151.5) | 138.7 (131.4-146.4) | 135.7 (130.1-141.6) | 132.8 (128.7-137.1) | 130.0 (126.9-133.2) | 127.2 (124.3-130.2) | 124.5 (120.9-128.2) | 121.8 (117.1-126.7) | 119.2 (113.3-125.4) | 116.0 (110.8-121.4) | 112.9 (108.4-117.6) | 109.8 (105.8-114.0) | 106.9 (103.1-110.8) | 104.0 (100.4-107.8) | 101.2 (97.5-105.1) | 98.5 (94.6-102.6) |
|  | Women | 227.5 (212.8-243.2) | 221.1 (208.9-234.0) | 214.9 (205.0-225.3) | 208.9 (201.1-216.9) | 203.0 (197.2-209.0) | 197.3 (193.0-201.7) | 191.8 (188.3-195.4) | 186.4 (182.7-190.3) | 181.2 (176.5-186.1) | 176.4 (172.6-180.3) | 171.7 (168.7-174.9) | 167.2 (164.6-169.9) | 162.8 (160.2-165.4) | 158.4 (155.6-161.4) | 154.3 (150.9-157.7) | 150.2 (146.2-154.2) |
| Stress and neurotic related disorder | All | 191.0 (183.0-199.4) | 183.5 (177.0-190.2) | 176.3 (171.1-181.7) | 169.4 (165.1-173.7) | 162.7 (159.0-166.6) | 156.3 (152.6-160.1) | 150.2 (146.1-154.4) | 144.3 (139.6-149.1) | 138.6 (133.3-144.2) | 133.6 (129.4-138.0) | 128.9 (125.5-132.3) | 124.2 (121.7-126.9) | 119.8 (117.6-122.0) | 115.5 (113.4-117.6) | 111.3 (108.9-113.8) | 107.4 (104.5-110.3) |
|  | Men | 144.9 (135.6-154.8) | 139.9 (132.7-147.5) | 135.0 (129.5-140.8) | 130.4 (126.0-135.0) | 125.9 (121.7-130.3) | 121.6 (116.7-126.6) | 117.4 (111.5-123.6) | 113.3 (106.3-120.9) | 109.4 (101.2-118.4) | 105.9 (99.0-113.3) | 102.5 (96.8-108.5) | 99.2 (94.4-104.3) | 96.0 (91.7-100.5) | 92.9 (88.6-97.4) | 89.9 (85.3-94.7) | 87.0 (81.9-92.4) |
|  | Women | 216.3 (207.0-226.0) | 207.5 (199.8-215.4) | 199.0 (192.8-205.4) | 190.9 (185.9-196.0) | 183.1 (179.0-187.3) | 175.6 (172.0-179.3) | 168.5 (164.9-172.1) | 161.6 (157.6-165.7) | 155.0 (150.4-159.7) | 149.2 (145.6-152.9) | 143.7 (140.9-146.5) | 138.3 (136.1-140.6) | 133.2 (131.3-135.1) | 128.2 (126.2-130.3) | 123.5 (121.2-125.8) | 118.9 (116.1-121.7) |
| Personality Disorder | All | 610.9 (593.5-628.9) | 584.9 (571.6-598.5) | 560.0 (550.2-569.9) | 536.1 (528.8-543.6) | 513.3 (506.7-520.0) | 491.5 (483.9-499.1) | 470.5 (461.1-480.2) | 450.5 (438.9-462.4) | 431.3 (417.6-445.5) | 416.8 (404.9-429.0) | 402.7 (392.0-413.7) | 389.1 (378.9-399.6) | 376.0 (365.7-386.6) | 363.3 (352.4-374.6) | 351.1 (339.1-363.4) | 339.2 (326.1-352.8) |
|  | Men | 298.4 (273.5-325.7) | 286.3 (266.4-307.7) | 274.6 (259.2-290.9) | 263.4 (251.8-275.7) | 252.7 (243.5-262.3) | 242.4 (233.8-251.3) | 232.5 (222.8-242.7) | 223.1 (211.3-235.5) | 214.0 (199.9-229.1) | 206.3 (194.3-219.0) | 198.9 (188.6-209.8) | 191.8 (182.6-201.4) | 184.9 (176.3-193.8) | 178.3 (169.7-187.2) | 171.9 (162.9-181.3) | 165.7 (156.0-176.0) |
|  | Women | 765.6 (747.4-784.2) | 733.9 (720.2-747.9) | 703.5 (693.1-714.0) | 674.4 (665.5-683.3) | 646.4 (637.1-655.9) | 619.7 (608.3-631.2) | 594.0 (580.2-608.1) | 569.4 (553.0-586.3) | 545.8 (526.9-565.4) | 527.7 (511.7-544.2) | 510.1 (495.9-524.7) | 493.1 (479.5-507.1) | 476.7 (462.6-491.2) | 460.8 (445.4-476.8) | 445.5 (428.2-463.5) | 430.7 (411.4-450.9) |
| ASD/ADHD | All | 130.5 (125.3-136.0) | 128.0 (123.8-132.4) | 125.6 (122.3-129.0) | 123.2 (120.6-125.9) | 120.8 (118.5-123.2) | 118.5 (116.0-121.1) | 116.3 (113.2-119.4) | 114.0 (110.3-117.9) | 111.8 (107.4-116.5) | 107.8 (104.2-111.6) | 103.9 (101.0-107.0) | 100.2 (97.7-102.7) | 96.6 (94.3-99.0) | 93.1 (90.6-95.6) | 89.8 (87.0-92.6) | 86.5 (83.3-89.9) |
|  | Men | 80.6 (75.8-85.6) | 78.9 (75.0-83.1) | 77.3 (74-80.7) | 75.7 (72.9-78.6) | 74.1 (71.5-76.8) | 72.6 (69.9-75.4) | 71.1 (68.1-74.2) | 69.6 (66.1-73.3) | 68.2 (64.2-72.5) | 65.4 (62.1-68.7) | 62.6 (59.9-65.5) | 60.0 (57.4-62.8) | 57.5 (54.6-60.6) | 55.1 (51.8-58.6) | 52.8 (49.0-56.9) | 50.6 (46.3-55.3) |
|  | Women | 198.3 (190.9-206.0) | 194.7 (188.8-200.7) | 191.1 (186.5-195.7) | 187.5 (184.1-191.1) | 184.1 (181.1-187.1) | 180.7 (177.5-183.9) | 177.3 (173.4-181.3) | 174.1 (169.2-179.1) | 170.8 (164.9-177.0) | 164.9 (159.9-170.1) | 159.2 (155.0-163.5) | 153.6 (150.1-157.3) | 148.3 (145.1-151.6) | 143.1 (140.0-146.3) | 138.2 (134.9-141.5) | 133.4 (129.8-137.1) |
| Other disorder | All | 119.3 (115.4-123.3) | 117.2 (114.1-120.4) | 115.1 (112.7-117.6) | 113.1 (111.2-115.0) | 111.1 (109.4-112.7) | 109.1 (107.3-110.9) | 107.1 (105.0-109.4) | 105.2 (102.6-108.0) | 103.4 (100.1-106.7) | 101.3 (98.6-104.2) | 99.3 (97.0-101.7) | 97.4 (95.3-99.4) | 95.5 (93.6-97.4) | 93.6 (91.7-95.5) | 91.7 (89.6-93.9) | 89.9 (87.5-92.3) |
|  | Men | 55.9 (50.3-62.0) | 56.2 (51.5-61.4) | 56.6 (52.7-60.8) | 57.0 (53.7-60.4) | 57.4 (54.5-60.4) | 57.7 (54.9-60.7) | 58.1 (54.9-61.5) | 58.5 (54.6-62.7) | 58.9 (54.1-64.0) | 57.4 (53.6-61.5) | 55.9 (52.9-59.1) | 54.5 (52.1-57.0) | 53.1 (51.0-55.4) | 51.8 (49.5-54.2) | 50.5 (47.7-53.5) | 49.2 (45.8-52.8) |
|  | Women | 174.7 (170.7-178.9) | 170.2 (166.9-173.6) | 165.8 (163.1-168.6) | 161.6 (159.2-164.0) | 157.4 (155.1-159.8) | 153.3 (150.8-155.9) | 149.4 (146.5-152.3) | 145.5 (142.2-149.0) | 141.8 (137.9-145.7) | 139.2 (135.9-142.6) | 136.7 (133.6-139.8) | 134.2 (131-137.5) | 131.8 (128.1-135.6) | 129.4 (125.0-133.9) | 127 (122-132.3) | 124.7 (118.9-130.9) |
| No mental disorder | All | 3.5 (3.4-3.7) | 3.5 (3.3-3.6) | 3.4 (3.3-3.5) | 3.3 (3.2-3.4) | 3.3 (3.2-3.4) | 3.2 (3.1-3.3) | 3.2 (3.0-3.3) | 3.1 (2.9-3.3) | 3.0 (2.8-3.3) | 3.0 (2.8-3.1) | 2.9 (2.8-3.0) | 2.8 (2.7-2.9) | 2.7 (2.7-2.8) | 2.7 (2.6-2.8) | 2.6 (2.5-2.7) | 2.5 (2.4-2.7) |
|  | Men | 3.1 (2.9-3.4) | 3.1 (2.9-3.3) | 3.0 (2.9-3.2) | 3.0 (2.8-3.1) | 2.9 (2.8-3.1) | 2.9 (2.7-3.1) | 2.8 (2.6-3.0) | 2.8 (2.6-3.0) | 2.7 (2.5-3.0) | 2.6 (2.4-2.9) | 2.5 (2.4-2.7) | 2.4 (2.3-2.5) | 2.3 (2.2-2.4) | 2.2 (2.1-2.4) | 2.2 (2.0-2.3) | 2.1 (1.9-2.3) |
|  | Women | 3.9 (3.8-4.1) | 3.9 (3.8-4.0) | 3.8 (3.7-3.9) | 3.7 (3.6-3.8) | 3.6 (3.6-3.7) | 3.6 (3.5-3.6) | 3.5 (3.4-3.6) | 3.4 (3.3-3.5) | 3.3 (3.2-3.5) | 3.3 (3.2-3.4) | 3.2 (3.1-3.4) | 3.2 (3.1-3.3) | 3.1 (3.0-3.2) | 3.1 (3.0-3.2) | 3.0 (2.9-3.2) | 3.0 (2.9-3.1) |

**Supplementary Figure 2:** Multivariate-adjusted* incidence rates with 95% confidence intervals of suicide attempt per 10,000 person-years during the years preceding the start of the Covid-19 pandemic (first quarter 2018 or 2018Q1 - 2020Q1) and the years after start of the pandemic (2020Q2 – 2021Q4) for individuals with specific severe mental disorders treated in specialised healthcare and individuals without such treated disorders the year preceding the start of the respective quarter, covering the entire population > 10 years of age in Sweden

*Adjusted for sex, age, educational level, family situation, country of birth, type of living area, previous sickness absence, disability pension and/or unemployment; ASD/ADHD - Austism spectrum disorders/Attention deficit hyperactivity disorder

Submitted as separate file

**Supplementary Table 4:** Multivariate-adjusted^1^ change in Incidence Rates (IR)^2^ before (first quarter 2018 or 2018Q1 - 2020Q1) and during the Covid-19 pandemic (2020Q2 – 2021Q4) and the ratio of change in IRs during versus before the pandemic^3^ of suicide attempt for groups of individuals with/without pre-existing severe mental disorders treated in specialised healthcare the year preceding the respective quarter.

|  | **Pre-Covid-19 pandemic** | | **Covid-19 pandemic** | | **Pre- vs Covid-19 pandemic** | |
| --- | --- | --- | --- | --- | --- | --- |
| **Pre-existing severe mental disorder** | **Change in IR (95% CI)** | **P-value** | **Change in IR (95% CI)** | **P-value** | **Ratio of change in IR (95% CI)** | **P-value** |
| Substance misuse | 0.96 (0.955-0.973) | <0.001 | 0.97 (0.958-0.979) | <0.001 | 1.00 (0.987-1.023) | 0.603 |
| Non-affective psychosis | 1.01 (0.990-1.029) | 0.360 | 0.99 (0.976-1.020) | 0.845 | 0.99 (0.953-1.026) | 0.544 |
| Depressive/Bipolar disorder | 0.98 (0.973-0.992) | <0.001 | 0.98 (0.965-0.990) | <0.001 | 0.99 (0.976-1.015) | 0.622 |
| Stress & neurotic-rel. disorders | 0.97 (0.966-0.982) | <0.001 | 0.97 (0.961-0.982) | <0.001 | 0.99 (0.980-1.014) | 0.718 |
| Personality disorder | 0.97 (0.959-0.983) | <0.001 | 0.97 (0.960-0.988) | <0.001 | 1.00 (0.981-1.027) | 0.779 |
| Other mental disorders | 0.99 (0.969-1.002) | 0.087 | 0.98 (0.961-0.997) | 0.022 | 0.99 (0.963-1.024) | 0.670 |
| ASD/ADHD^4^ | 0.99 (0.972-1.007) | 0.223 | 0.97 (0.949-0.987) | 0.001 | 0.98 (0.947-1.011) | 0.198 |
| No mental disorders | 0.98 (0.973-0.997) | 0.017 | 0.97 (0.960-0.990) | 0.001 | 0.99 (0.966-1.015) | 0.429 |

^1^Adjusted for sex, age, educational level, family situation, country of birth, type of living area, previous sickness absence, disability pension and/or unemployment
^2^Slopes based on the GEE models with a linear fit showing the % change in IR for one unit (quarter) change during the pre- and pandemic periods.
^3^Estimates of slopes during the pandemic divided by the estimates of slopes before the pandemic.
^4^Autism spectrum disorders/Attention deficit hyperactivity disorders.
